# Soluble Neuropilin-1 Response to Hypoglycemia in Type 2 Diabetes: Increased Risk or Protection in SARS-CoV-2 Infection?

**DOI:** 10.3389/fendo.2021.665134

**Published:** 2021-06-23

**Authors:** Abu Saleh Md Moin, Ahmed Al-Qaissi, Thozhukat Sathyapalan, Stephen L. Atkin, Alexandra E. Butler

**Affiliations:** ^1^ Diabetes Research Center (DRC), Qatar Biomedical Research Institute (QBRI), Hamad Bin Khalifa University (HBKU), Qatar Foundation (QF), Doha, Qatar; ^2^ Academic Endocrinology, Diabetes and Metabolism, Hull York Medical School, Hull, United Kingdom; ^3^ Department of Endocrinology, Leeds Medical School, Leeds, United Kingdom; ^4^ Department of Research, Royal College of Surgeons in Ireland Bahrain, Adliya, Bahrain

**Keywords:** Neuropilin-1, type 2 diabetes, COVID-19, SARS-CoV-2, ACE inhibitors, ADAM9

## Abstract

**Introduction:**

Neuropilin-1(NRP1) is a cofactor that enhances SARS-CoV-2 coronavirus cell infectivity when co-expressed with angiotensin-converting enzyme 2(ACE2). The Renin-Angiotensin System (RAS) is activated in type 2 diabetes (T2D); therefore, the aim of this study was to determine if hypoglycaemia-induced stress in T2D would potentiate serum NRP1(sNRP1) levels, reflecting an increased risk for SARS-CoV-2 infection.

**Methods:**

A case-control study of aged-matched T2D (n = 23) and control (n = 23) subjects who underwent a hyperinsulinemic clamp over 1-hour to hypoglycemia(<40mg/dl) with subsequent timecourse of 4-hours and 24-hours. Slow Off-rate Modified Aptamer (SOMA)-scan plasma protein measurement determined RAS-related proteins: renin (REN), angiotensinogen (AGT), ACE2, soluble NRP1(sNRP1), NRP1 ligands (Vascular endothelial growth factor, VEGF and Class 3 Semaphorins, SEM3A) and NRP1 proteolytic enzyme (A Disintegrin and Metalloproteinase 9, ADAM9).

**Results:**

Baseline RAS overactivity was present with REN elevated and AGT decreased in T2D (p<0.05); ACE2 was unchanged. Baseline sNRP1, VEGF and ADAM9 did not differ between T2D and controls and remained unchanged in response to hypoglycaemia. However, 4-hours post-hypoglycemia, sNRP1, VEGF and ADAM9 were elevated in T2D(p<0.05). SEMA3A was not different at baseline; at hypoglycemia, SEMA3A decreased in controls only. Post-hypoglycemia, SEMA3A levels were higher in T2D versus controls. sNRP1 did not correlate with ACE2, REN or AGT. T2D subjects stratified according to ACE inhibitor (ACEi) therapies showed no difference in sNRP1 levels at either glucose normalization or hypoglycaemia.

**Conclusion:**

Hypoglycemia potentiated both plasma sNRP1 level elevation and its ligands VEGF and SEMA3A, likely through an ADAM9-mediated mechanism that was not associated with RAS overactivity or ACEi therapy; however, whether this is protective or promotes increased risk for SARS-CoV-2 infection in T2D is unclear.

**Clinical Trial Registration:**

https://clinicaltrials.gov, identifier NCT03102801.

## Introduction

Neuropilin-1 (NRP1) is a cofactor that enhances SARS-CoV-2 coronavirus cell infectivity when co-expressed with ACE2 (1). SARS-CoV-2 uses the spike protein for cell entry, and its cleavage facilitates attachment to NRP1; therefore, tissues with increased membrane NRP1 have increased infectivity risk (1) and increased circulating NRP1 expression may raise infection risk. NRP1 is a 120–130 kDa surface-expressed glycoprotein, initially characterized as a neuronal receptor for specific secreted members of the semaphorin family (SEMA3) involved in axon repulsion (2). NRP-1 also serves as a receptor for a number of isoforms of vascular endothelial growth factor (VEGF). Soluble isoforms of NRP1 (sNRP1) also exist without transmembrane or cytoplasmic domains and function as natural ligand sequesters, inhibiting the interaction of VEGF-A or other growth factors with their specific receptors and with membrane-bound NRP1 (3). The generation of sNRP1 is mediated *via* proteolytic cleavage by a disintegrin or metalloproteinase-9 or 10 (ADAM9 or ADAM10) (4). NRP1 interacts with the RAS, a risk factor for COVID-19 disease (5), contributing to protection from angiotensin II-induced hypertension (6).

T2D is associated with high risk for acquiring SARS-Cov-2 infection, severe disease, acute respiratory distress syndrome and increased mortality (7). Diabetic patients have an overactive RAS with ACE2 being overexpressed in kidney (8) and the circulation (9), and ACE2 expression may be increased in lungs that is thought to increase susceptibility and severity to SARS-Cov-2 infection. Furthermore, patients with T2D exist in a state of chronic low-grade inflammation (10); both hypoglycaemia and hyperglycemia compound the risk of worse outcomes in hospitalized T2D patients with COVID-19 (11), likely due to the increase in inflammatory mediators that further promote risk for an acute cardiovascular event and/or multi-organ failure (12, 13).

Here, we hypothesized that underlying RAS activation may potentiate the levels of sNRP1 and its ligands (VEGF and SEMA3A) in hypoglycaemia-induced stress in type 2 diabetes (T2D), reflecting an increased risk for SARS-CoV-2 infection.

## Materials and Methods

### Study Design

This prospective parallel study was performed in 46 subjects, adult T2D (n=23) and control (n=23). The duration of diabetes was <10 years and all T2D subjects were on a stable dose of medication (metformin, statin and/or angiotensin converting enzyme inhibitor/angiotensin receptor blocker) over the prior 3 months. For those with T2D, no medications for glycemic control except metformin were allowed. The study was performed at the Diabetes Centre at Hull Royal Infirmary. All participants provided written informed consent. The trial was approved by the North West-Greater Manchester East Research Ethics Committee (REC number:16/NW/0518), registered at www.clinicaltrials.gov (NCT03102801) and conducted according to the Declaration of Helsinki.

### Hyperinsulinemic Clamp

**Figure 1:**
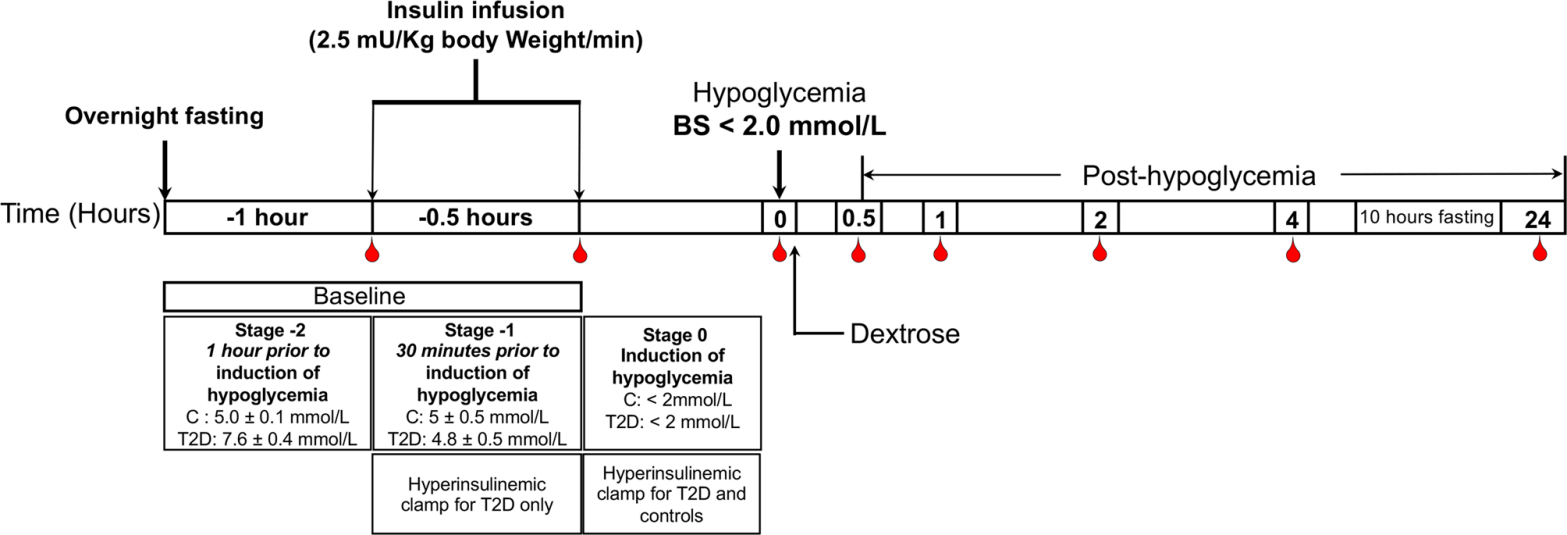
Schematic diagram of the insulin clamp study, showing the intervention and blood sampling time points.

The method for performing the insulin clamp has been published previously (14). A schematic diagram of the insulin clamp study, showing the intervention and blood sampling time points has been outlined in **Figure 1**. Briefly, after an overnight fast, bilateral ante-cubital fossa indwelling cannulae were inserted 30 to 60 minutes prior to the commencement of the clamp (8:30 AM). To induce hypoglycemia, soluble intravenous insulin (Humulin S; Eli Lilly, Liverpool. UK) was given in a pump starting at a dose of 2.5 mU/Kg body weight (BW)/min, with an increment of 2.5mU/Kg BW/min every 15 minutes until two readings of venous blood glucose measured by a glucose analyser (HemoCue glucose 201+, Sweden) of 2.2 mmol/L (<40 mg/dl) or a single reading of 2.0 mmol/L (36 mg/dL) was obtained (14). The blood sample schedule was timed subsequently with respect to the time point when hypoglycemia occurred (**Figure 1**). Following the identification of hypoglycemia, intravenous glucose was given in the form of 150 mL of 10% dextrose and repeat blood glucose checks were performed after 5 minutes if blood glucose was still <4.0 mmol/L. All patients achieved a blood glucose of 2.0 mmol/L (36 mg/dL) at the point of hypoglycemia.

### Blood Sample Preparation and Biochemical Marker Analyses

Venous blood samples collected during the screening visit were analysed for serum insulin, total cholesterol, triglycerides, HDL cholesterol, C-reactive protein (CRP) and glycated haemoglobin (HbA1C).

Blood samples were separated immediately by centrifugation at 3500g for 15 minutes at 4°C, and the aliquots were stored at –80°C, within 30-minutes of blood collection, until batch analysis. Serum insulin was assayed using a competitive chemiluminescent immunoassay performed on the manufacturer’s DPC Immulite 2000 analyser (Euro/DPC, Llanberies, UK), with a coefficient of variation of 6 and no stated cross-reactivity with proinsulin. Fasting plasma glucose (FPG), total serum cholesterol, triglycerides, and high-density lipoprotein (HDL) cholesterol levels were measured enzymatically using a Beckman AU 5800 analyser (Beckman-Coulter, High Wycombe, UK). LDL cholesterol was calculated using the Friedewald equation. Plasma whole blood samples were analysed for HbA1C on a Menarini Diagnostics HB9210 premier (A. Menarini Diagnostics Ltd, Winnersh-Wokingham, UK).

### SOMA-Scan Assay

The SOMAscan assay used to quantify proteins was performed on an in-house Tecan Freedom EVO liquid handling system (Tecan Group, Maennedorf, Switzerland) utilizing buffers and SOMAmers from the SOMAscan HTS Assay 1.3K plasma kit (SomaLogic, Boulder, CO) according to manufacturer’s instructions and as described previously (15–17). Initial Relative Fluorescent Units (RFUs) were obtained from microarray intensity images using the Agilent Feature Extraction Software (Agilent, Santa Clara, CA). Raw RFUs were normalized and calibrated using the software pipeline provided by SomaLogic.

Statistical analyses were performed on log_2_ RFU values using R version 3.5.2 (R Foundation for Statistical Computing, Vienna, Austria) including base R package. Data handling and differential protein expression were analyzed using the autonomics and limma (18) packages. For differential protein analysis we applied limma models containing contrasts between timepoints, as well as contrasts between healthy and patients with diabetes at single timepoints. In both models, blocking by patient ID was performed to account for random effects. Batch effect correction was performed by adding batch as a covariate to the model. Limma obtained P values were corrected using the Benjamini-Hochberg method (19).

### Statistical Analysis

There are no studies detailing the changes in NRP1 proteins in response to hypoglycaemia on which to base a power calculation. Sample size for pilot studies has been reviewed by Birkett and Day (20). They concluded that a minimum of 20 degrees-of-freedom was required to estimate effect size and variability. Hence, we needed to analyse the samples from a minimum of 20 patients per group. Data trends were visually evaluated for each parameter and non-parametric tests were applied on data that violated the assumptions of normality when tested using the Kolmogorov-Smirnov Test. Comparison between groups was performed at each timepoint using Student’s t-test. A p-value of <0.05 was considered statistically significant. Statistical analysis was performed using Graphpad Prism (San Diego, CA, USA).

## Results

### Study Participants

T2D (n=23) and control (n=23) subjects were matched for age (p=ns); T2D had higher BMI (p=0.0012); duration of disease was 4.5 ± 2.9 years (**Table 1**). Nine T2D subjects were treated with ACE inhibitor (ACEi) therapy. Systolic and diastolic blood pressure were higher in T2D (p<0.001). Renin was elevated and angiotensinogen decreased in T2D (p<0.05), indicating RAS overactivity; ACE2 was unchanged (**Table 2**) (5).

**Table 1:** Demographic and clinical characteristics of the study participants.

Baseline	Type 2 Diabetes (n = 23)	Controls (n = 23)	p-value
Age (years)	64 ± 8	60 ± 10	<0.0001
Sex (M/F)	12/11	11/12	0.77
Weight (kg)	90.9 ± 11.1	79.5 ± 8.8	<0.0001
Height (cm)	167 ± 14	169 ± 5	0.64
BMI (kg/m^2^)	32 ± 4	28 ± 3	<0.0001
Systolic BP (mmHg)	132 ± 8	122 ± 8	0.001
Diastolic BP (mmHg)	81 ± 7	75 ± 6	0.003
Duration of diabetes (years)	4.5 ± 2.2	N/A	
HbA1c (mmol/mol)	51.2 ± 11.4	37.2 ± 2.2	<0.0001
HbA1c (%)	6.8 ± 1.0	5.6 ± 0.2	<0.0001
Total cholesterol (mmol/l)	4.2 ± 1.01.0	4.8 ± 0.77	0.02
Triglyceride (mmol/l)	1.7 ± 0.7	1.34 ± 0.6	0.06
HDL-cholesterol (mmol/l)	1.1 ± 0.3	1.5 ± 0.4	0.001
LDL-cholesterol (mmol/l)	2.23 ± 0.8	2.7 ± 0.87	0.051
CRP (mg/l)	3.10 ± 2.87	5.30 ± 1110.03	0.66

BMI, Body mass index; BP, Blood pressure; HDL-cholesterol, High density lipoprotein cholesterol; LDL-cholesterol, Low density lipoprotein cholesterol; CRP, C-reactive protein; HbA1c, Hemoglobin A1c.

**Table 2:** Circulating levels of renin angiotensin system (RAS)-related proteins at baseline and in response to hypoglycemia in control subjects and in subjects with T2D.

T2D subjects					
Renin (RFU)		Angiotensinogen (RFU)		ACE2 (RFU)	
Baseline (BL)	Hypoglycemia (H)	Baseline (BL)	Hypoglycemia (H)	Baseline (BL)	Hypoglycemia (H)
1730 ± 566	1601 ± 511	3786 ± 174	4027 ± 261	285 ± 29	286 ± 29
P=0.5 (BL *vs* H in T2D)		P=0.7 (BL *vs* H in T2D)		P=0.9 (BL *vs* H in T2D)	
Control subjects					
Renin (RFU)		Angiotensinogen (RFU)		ACE2 (RFU)	
Baseline (BL)	Hypoglycemia (H)	Baseline (BL)	Hypoglycemia (H)	Baseline (BL)	Hypoglycemia (H)
675 ± 71	666 ± 70	5005 ± 573	5248 ± 558	281 ± 18	277 ± 17
P=0.5 (BL *vs* H in control)		P=0.5 (BL *vs* H in control)		P=0.8 (BL *vs* H in control)	
**P=0.02 (T2D *vs* control at BL)**		**P=0.04 (T2D *vs* control at BL)**		P=0.7 (T2D *vs* control)	

Data is expressed as Mean ± SEM. RFU, relative fluorescent units; BL, baseline; H, hypoglycemia.

### Changes of Plasma sNRP1 Levels in Response to Glucose Normalization and Hypoglycemia

**Figure 2:**
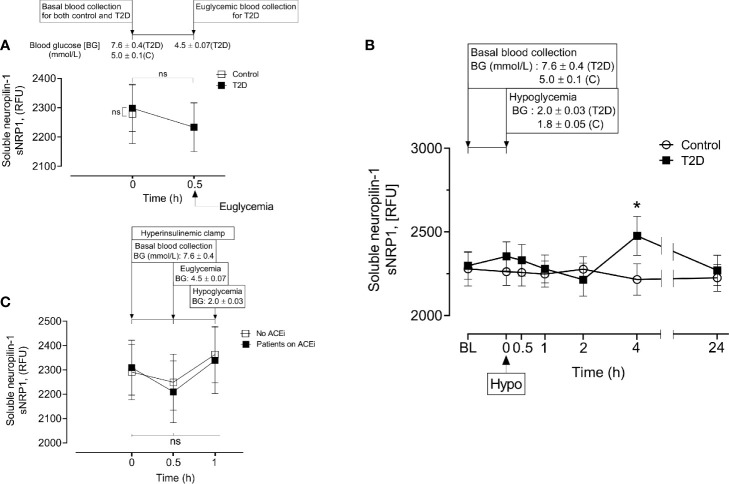
Circulatory levels of Neuropilin-1 (soluble neuropilin-1, sNRP1) in plasma before, during and after iatrogenic induction of hypoglycemia. Blood sampling was performed at baseline (BL), euglycemia (30 min before hypoglycemia in type 2 diabetes (T2D) only), at hypoglycemia (0 min) and post-hypoglycemia (30 minutes, 1-hour, 2-hours, 4-hours and 24-hours). At baseline (BL), blood glucose (BG) was 7.6 ± 0.4 mmol/L (for T2D) and 5.0 ± 0.1 mmol/L (for control, **C**). At glucose normalization, BG was 4.5 ± 0.07 mmol/L (for T2D). At the point of hypoglycemia, BG was 2.0 ± 0.03 mmol/L (for T2D) and 1.8 ± 0.05 mmol/L (for control). **(A)** Effect of glucose normalization on plasma sNRP1 levels in T2D (black squares) and control (open squares) subjects. **(B)**, Changes in sNRP1 levels in response to hypoglycaemia and post-hypoglycemic timepoints in control (white circles) and in T2D (black squares) subjects. **(C)**, Effect of glucose normalization and hypoglycaemia on plasma levels of sNRP1 in T2D patients who were treated with ACE inhibitors (ACEi) (black squares) and those without ACEi therapy (white squares). *p < 0.05, T2D *vs* control; Hypo, hypoglycaemia; RFU, relative fluorescent units; ns, not significant.

**Figure 3:**
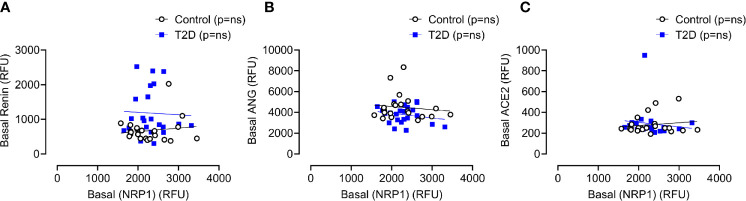
Correlation of plasma levels of soluble NRP1 (sNRP1) with basal levels of renin angiotensinogen system (RAS) proteins. Correlation of soluble NRP1 (sNRP1) with Renin **(A)**, angiotensinogen (ANG) **(B)** and angiotensin-converting enzyme 2 (ACE2) **(C)**. No correlation of sNRP1 with these RAS proteins was found in either T2D or control subjects. Control subjects, open black circles; T2D subjects, solid blue squares; ns, not significant.

Baseline levels of sNRP1 did not differ between T2D and controls (2298 ± 385 *vs* 2279 ± 488 RFU, T2D *vs* control, p=ns); normalization of glucose in T2D did not alter sNRP1 (2298.1 ± 80.3 *vs* 2279.1 ± 101.6 RFU of NRP1, T2D *vs* control, p=ns) (**Figure 2A**), and levels were unchanged in response to insulin induced normalization of glycemia (in T2D) (**Figure 2A**) and hypoglycaemia (both in control and in T2D) (**Figure 2B**). However, 4-hours post-hypoglycemia, NRP1 was elevated in T2D (2476 ± 117 *vs* 2216 ± 94 RFU, T2D *vs* control, p=0.04) (**Figure 2B**). When T2D subjects were stratified according to ACE inhibitor (ACEi) therapies, there was no difference in sNRP1 levels in subgroups at either glucose normalization or hypoglycaemia (**Figure 2C**). sNRP1 did not correlate with any of the renin angiotensin system (RAS) proteins measured here: ACE2, REN or AGT (**Figure 3**).

### Changes of Plasma Levels of NRP1 Proteolytic Enzyme ADAM9 in Response to Hypoglycemia in T2D

**Figure 4:**
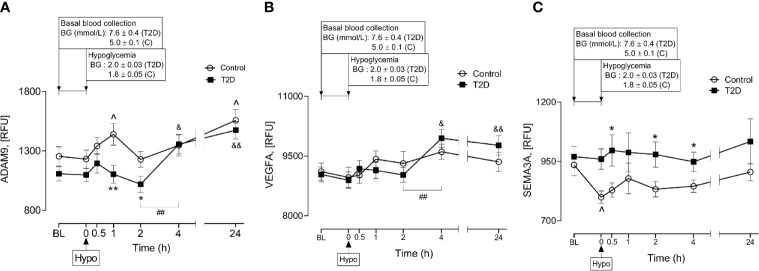
Plasma levels of A Disintegrin and Metalloproteinase 9 (ADAM9), class 3 semaphorins (SEMA3A) and vascular endothelial growth factor (VEGF) before, during and after iatrogenic induction of hypoglycemia. Changes in plasma ADAM9 **(A)**, VEGF **(B)** and SEMA3A **(C)** levels in response to hypoglycaemia and post-hypoglycemic timepoints in control (white circles) and T2D (black squares) subjects. *p < 0.05, **p < 0.01, control *vs* T2D; ^p < 0.05, control hypoglycaemia *vs* control post-hypoglycemia timepoints; ^&^p < 0.05, ^&&^p < 0.01, T2D hypoglycaemia *vs* T2D post-hypoglycemia timepoints; ^##^p < 0.01, T2D 2-h post-hypoglycemia *vs* T2D 4-h post-hypoglycemia. Hypo, hypoglycemia; RFU, relative fluorescent units.

A Disintegrin and Metalloproteinase 9 (ADAM9) has been reported to be involved in proteolysis of NRP1 in human endothelial cells and that this process was stimulated by vascular endothelial growth factor (VEGF) (4). Therefore, plasma ADAM9 levels were determined and showed that ADAM9 did not differ at baseline between control and T2D subjects (1107 ± 60 *vs* 1255 ± 81 RFU of ADAM9, T2D *vs* control, p=ns) but were decreased following hypoglycaemia in T2D (1h and 2h post-hypoglycemia) (**Figure 4A**). Interestingly, in T2D, ADAM9 levels increased sharply 4-hours post-hypoglycemia compared to either the 2h post-hypoglycemia timepoint (1356 ± 81 *vs* 1019 ± 68 RFU of ADAM9 4h post-hypo *vs* 2h post-hypo, p<0.01) or baseline (1356 ± 81 *vs* 1107 ± 60 RFU of ADAM9 4h post-hypo *vs* baseline, p<0.05) (**Figure 4A**). Elevated ADAM9 levels were also observed 24h post-hypoglycemia both in control and T2D subjects (**Figure 4A**).

### Changes of NRP1 Ligands (VEGFA and SEMA3A) in Response to Hypoglycaemia in T2D

Soluble isoforms of NRP1 (sNRP1) also exist without transmembrane or cytoplasmic domains, expressing only the extracellular domains which allow them to bind NRP1 ligands (21); therefore, the plasma levels of NRP1 ligands (VEGFA and SEMA3A) were determined in response to insulin-induced hypoglycemia. Neither VEGFA nor SEMA3A was different at baseline in T2D compared to controls (9037 ± 188 *vs* 9113 ± 212 RFU of VEGF, T2D *vs* control, p=ns; 970 ± 43 *vs* 935 ± 45 RFU of SEMA3A, T2D *vs* control, p=ns). Normalization of hyperglycemia did not alter plasma VEGF or SEMA3A levels in T2D (9145 ± 275 *vs* 9037 ± 188 RFU of VEGF, euglycemia *vs* baseline in T2D, p=ns; 965 ± 51 *vs* 970 ± 43, RFU of SEMA3A, euglycemia *vs* baseline in T2D, p=ns). While insulin-induced hypoglycemia did not alter plasma VEGF levels in either control or T2D subjects (**Figure 4B**), plasma SEMA3A levels significantly decreased at hypoglycemia in control cases compared to baseline (799 ± 27 *vs* 935 ± 45 RFU of SEMA3A in control, hypoglycemia *vs* baseline, p=0.01) (**Figure 4C**). Post-hypoglycemic SEMA3A levels (from 0.5h post-hypo to 24h post-hypo) did not differ from baseline or hypoglycemia in either controls or T2D; however, SEMA3A levels were generally elevated in T2D, showing significance at 0.5h, 2h and 4h post-hypoglycemia (p<0.05) compared to controls (**Figure 4C**). Interestingly, in a similar manner to ADAM9, VEGF levels also increased sharply 4-hours post-hypoglycemia compared to the 2h post-hypoglycemia, hypoglycemia or baseline timepoints (4h *vs* 2h: 9947 ± 225 *vs* 9026 ± 185 RFU of VEGF, p=0.002; 4h *vs* hypoglycemia: 9947 ± 225 *vs* 8894 ± 208 RFU of VEGF, p=0.001; 4h *vs* baseline: 9947 ± 225 *vs* 9037 ± 188 RFU of VEGF, p=0.003) (**Figure 4B**).

### Plasma RAS and sNRP1 Related Proteins in Response to Hyperglycemia in T2D

To determine whether hyperglycemia affects the levels of RAS or sNRP1 associated proteins, we performed correlation analysis between the change in blood glucose from hyperglycemia (baseline) to normoglycemia with basal levels of plasma RAS-related proteins (Renin, ANG and ACE2) (**Supplementary Figure 1A**) and plasma sNRP1 related proteins (sNRP1, VEGF, SEMA3A and ADAM17) (**Supplementary Figure 1B**). Our data indicate that none of the proteins differed in response to hyperglycemia in T2D.

### Gender Stratification

We next sought to determine whether sNRP1 related proteins differ between males and females in response to changes in glycemia. Stratification of plasma levels of sNRP1, VEGF, SEMA3A and ADAM17 based on gender demonstrated no significant differences in their levels at baseline or hypoglycemia in control subjects (**Supplementary Figures 2A, B**); nor at baseline, normoglycemia, or hypoglycemia in T2D subjects (**Supplementary Figures 2C–E**).

## Discussion

Here we report the levels of soluble neuropilin-1 (sNRP1), NRP1 proteolytic enzyme ADAM9 and NRP1 ligands, VEGF and SEMA3A, in response to insulin-induced hypoglycemia in obese patients with T2D. Our data demonstrate that soluble sNRP1, VEGF or SEMA3A and ADAM9 levels did not differ from control subjects in the basal condition in obese T2D; however, an alteration of their levels was observed in response to insulin-induced hypoglycemia. An elevation of sNRP1 levels in association with its proteolytic enzyme as well as ligands post-hypoglycemia suggests the possible connection of glycemic control with NRP1 cleavage in obese T2D subjects.

NRP1, a transmembrane glycoprotein, was initially identified as a receptor for class 3 semaphorins (SEMA3A), which are negative mediators of neuronal guidance (2, 22). NRP1 also functions as a high-affinity co-receptor for a number of vascular endothelial growth factor (VEGF) isoforms, in particular VEGF_165_, and enhances its activity in functions such as endothelial cell migration (23, 24). NRP1 is a single spanning transmembrane glycoprotein consisting of a large extracellular domain, a very short transmembrane domain and a short cytoplasmic domain. The NRP1 extracellular domain is divided into: (i) an A domain consisting of two a-domain repeats (a1a2) homologous to the complement proteins C1r and C1s, (ii) a B domain consisting of two b-domain repeats (b1b2) (25). In addition to the membrane form, a naturally occurring soluble NRP-1 protein (sNRP1) containing only the extracellular a1/a2 and b1/b2 domains is generated by alternative splicing of the NRP-1 gene (26) and this is capable of binding VEGF_165_ and SEMA3A (27). Therefore, sNRP1 is thought to function as a natural inhibitor of the membrane NRP1 by sequestering its ligands (3). Our data also demonstrate a simultaneous increase of sNRP1 and VEGF (4h post-hypoglycemia), suggesting a possible sNRP1 and VEGF interaction after the induction of hypoglycemia in obese T2D. sNRP1-induced ligand sequestration (by binding with VEGF) may block the interaction of VEGF and membrane-bound NRP1 (21); therefore, this action may reduce angiogenesis in severe COVID-19 disease and decrease the risk of SARS-CoV-2 cellular infectivity. Additionally, akin to sNRP1 antagonism of VEGF165 as an antiangiogenic agent (3), sNRP1 may bind to SARS-CoV-2 virus, preventing its binding to tissue NRP1 thus hindering or preventing its tissue entry.

There is evidence to suggest that increased sNRP1, seen here following insulin-induced transient hypoglycaemia but also as a consequence of strict glycemic control, might offer protection against COVID-19 disease severity. Optimizing glycemic control during hospitalization has been associated with a reduction in the risk of severe disease and death in patients with COVID-19 (28). Moreover, in a subset comparison of age and risk-matched COVID-19 patients with pre-existing T2D and poor glucose control (blood glucose >10.0 mmol/L), there was a reduction in mortality from 11.1% to 1.1% with significantly lower d-dimer, C-reactive protein (CRP) and interleukin 6 (IL6) when glucose was maintained in a range of 3.9-10 mmol/L (29). Since our data also demonstrated an increased plasma sNRP1 level 4-h post insulin-induced hypoglycaemia, it is highly likely that glycemic control-mediated improvement of severity in COVID-19 patients is associated with elevated sNRP1. However, further studies with blood sampling from COVID-19 patients should be performed to measure sNRP1 levels and to study the correlation between sNRP1 and COVID-19 risk factors (for example, CRP, d-dimer and IL6) in response to glycemic control in order to elucidate the mechanism.

The mechanism increasing sNRP1 levels in obese T2D in response to hypoglycemia is unclear; however, post-hypoglycemic ADAM9 levels were also higher at similar timepoints as the elevated sNRP1 levels in T2D cases. ADAM9 is a disintegrin and metalloproteinase involved in a wide array of cellular processes, especially those involving cell to cell interactions, adhesion, cell-matrix interactions, growth factor and cytokine signalling (30–32). Membrane-bound NRP1 is proteolytically cleaved by ADAM9 (or ADAM10) to produce its soluble form, sNRP1 (4). Here, ADAM9 was increased at 4-hours post-hypoglycemia in T2D and at 24-hours post-hypoglycemia in both T2D and control subjects. This suggests that ADAM9-mediated shedding of membrane bound NRP1 may potentiate elevated levels of sNRP1 over a prolonged period of time following a hypoglycaemic event.

Our data also demonstrate post-hypoglycemic elevation of plasma SEMA3A in T2D. SEMA3A levels have been reported to be significantly elevated in the vitreous fluid of patients with diabetic macular edema (33) and proliferative diabetic retinopathy (PDR) (34) *via* NRP1. Serum SEMA3A levels have also correlated with the phenotypes of diabetic retinopathy (35). Since secreted semaphorins (SEMA3 class) generally require NRPs as obligate co-receptors to interact with their surface receptors (36), it is likely that sNRP1 would also interact with SEMA3A in obese T2D in response to hypoglycemia. Thus, hypoglycemia-induced sNRP1 may pose a risk for diabetic microvascular complications which, in turn, may enhance the poor outcome of COVID19 severity in obese patients with T2D. However, apart from the connection of sNRP1 and risk of SARS-Cov-2, hypoglycemia-induced elevation in sNRP1 might also indicate a possible link between glucose counter-regulation and VEGF-NRP1 signalling in liver and kidney in T2D because sNRP1, but not NRP1, mRNA is expressed in liver hepatocytes and kidney distal and proximal tubules. On the other hand, NRP1 but not sNRP1, is expressed in liver veins and glomerular capillaries (3).

This study further showed that, whilst plasma renin was elevated, and angiotensinogen suppressed in T2D (5), that there were comparable levels of ACE2 and sNRP1 proteins between T2D and control subjects. In T2D, the sNRP1 levels did not change in response to normoglycemia or hypoglycemia regardless of whether or not the T2D subjects were on antihypertensive medication (ACEi). This suggests that there is RAS overactivation in T2D, and this was not affected by either acute normoglycemia or hypoglycaemia. However, sNRP1 levels were elevated post-hypoglycemia in T2D, suggesting that this hypoglycemic insult may result in a delayed but increased SARS-CoV-2 susceptibility. However, it is not precisely known how long that potential susceptibility window may last as blood sampling was not undertaken between the 4-hour and 24-hour post-hypoglycemia timepoints.

Whilst hyperglycemia may lead to more severe COVID-19 disease, the risk of hypoglycemia has also been shown to be increased during the COVID-19 pandemic, especially for those patients on the hypoglycemic agents sulphonylureas and insulin (37) and, paradoxically, that may mean that those T2D patients with the tightest, most optimal control (but with greater risk of hypoglycaemia) may be at higher risk of infection. Hydroxychloroquine may induce hypoglycemia in subjects with and without diabetes (38) and it has been shown that prophylactic hydroxychloroquine (HCQ) increases hypoglycemia in T2D (37); this may, therefore, conversely increase the subsequent risk of SARS-CoV-2 susceptibility. Moreover, an Indian study of patients with T2D revealed that the COVID-19 lockdown has been shown to increase the risk of hypoglycemia in patients with T2D, especially those receiving sulfonylureas (SU), insulin and HCQ, and especially in patients with associated co-morbidities (37). Notably, diabetic kidney disease (DKD), a co-morbidity already associated with increased risk of hypoglycaemia in non-COVID-19 infected patients due to factors such as reduced insulin clearance and degradation (39, 40), was the co-morbidity most frequently associated with hypoglycaemia during lockdown (37).

Study strengths include that these T2D subjects had a short disease duration and were relatively medication naïve. Study limitations include the small population under study and that measurement of circulating sNRP1, ADAM9, VEGF and SEMA3A may not reflect tissue levels; also, correlation of sNRP1 was with renin proteins rather than renin activity. In addition, the SOMAscan assay is designed as a discovery platform and measures relative protein concentrations using only external controls. Without internal controls and standard curves, it remains unclear which measurements are within the linear dynamic range (41); therefore, from a population point of view the same trends and associations will be present, but validation would be needed to individualise the results for treatment that was not done for the proteins reported here.

In conclusion, this data shows that strict blood glucose control associated with increased hypoglycemia potentiates sNRP1 levels *via* a mechanism involving ADAM9-mediated proteolytic cleavage of NRP1 that may continue to enhance sNRP1 levels following the hypoglycemic episode, independent of RAS activation or ACEi therapy. This post-hypoglycemia elevation of plasma sNRP1 may place patients with pre-existing obesity and T2D at increased risk for severe COVID19 disease; however, studies to clarify the role of sNRP1 in COVID19 patients are needed to determine if it promotes risk or affords protection for SARS-CoV-2 infection in T2D.

## Data Availability Statement 

The raw data supporting the conclusions of this article will be made available by the authors, without undue reservation.

## Ethics Statement 

The studies involving human participants were reviewed and approved by North West-Greater Manchester East Research Ethics Committee. The patients/participants provided their written informed consent to participate in this study.

## Author Contributions

AM and AB analyzed the data and wrote the manuscript. AA-Q contributed to study design, performed experiments, collected, analyzed, and interpreted data and edited the manuscript. TS supervised clinical studies and edited the manuscript. SA contributed to study design, data interpretation and the writing of the manuscript. AB is the guarantor of this work. All authors contributed to the article and approved the submitted version.

## Conflict of Interest

The authors declare that the research was conducted in the absence of any commercial or financial relationships that could be construed as a potential conflict of interest.

## Supplementary Material

Supplementary Figure 1 Correlation of change of blood glucose from normoglycemia to hyperglycemia with basal levels of renin angiotensinogen system (RAS) proteins, Renin, angiotensinogen (ANG) and angiotensin-converting enzyme 2 (ACE2) **(A)** and sNRP1 related proteins, sNRP1, VEGF, SEMA3A and ADAM17 **(B)** in T2D. No correlation of hyperglycemia with those proteins was found T2D subjects. Part A: Renin, open black circles; angiotensinogen (ANG), open blue squares; ACE2, open red diamonds. Part B: NRP1, open black circles; SEMA3A, open blue squares; ADAM9, open red diamonds; VEGF, open green triangles.

Supplementary Figure 2 Gender stratification of RAS proteins or sNRP1 related proteins at baseline or at hypoglycemia. No significant differences between male and female in the levels of sNRP1, VEGF, SEMA3A or ADAM17 at baseline and hypoglycemia in control **(A, B)**; and at baseline, normoglycemia and hypoglycemia in T2D **(C–E)**.
