# Supplementary figures and images for: Soluble Neuropilin-1 Response to Hypoglycemia in Type 2 Diabetes: Increased Risk or Protection in SARS-CoV-2 Infection?
Source: Front Endocrinol (Lausanne). 2021 Jun 23;12:665134. doi: 10.3389/fendo.2021.665134 (PMC8261232; doi:10.3389/fendo.2021.665134)

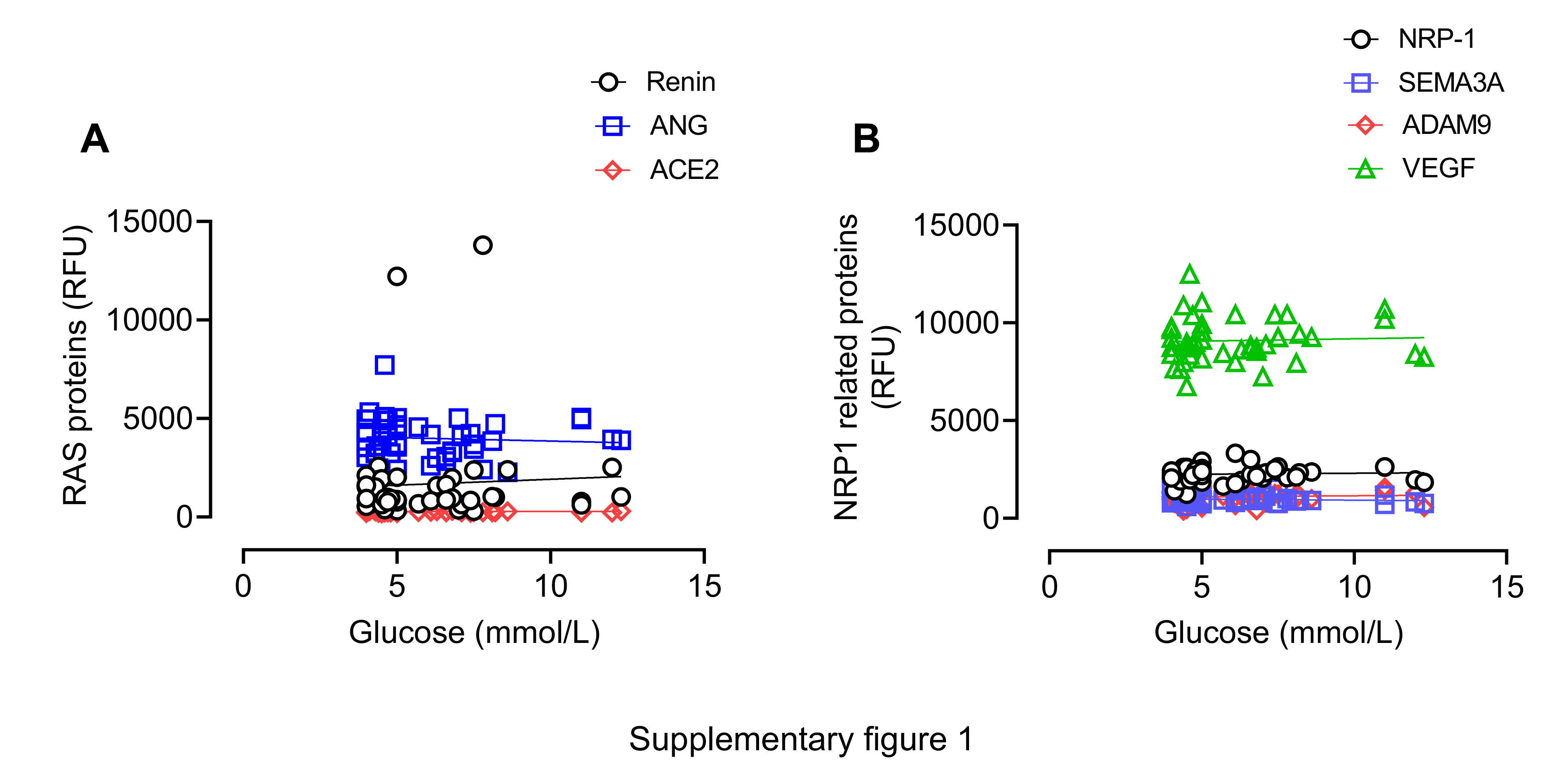

Supplement: Supplementary Figure 1 — Correlation of change of blood glucose from normoglycemia to hyperglycemia with basal levels of renin angiotensinogen system (RAS) proteins, Renin, angiotensinogen (ANG) and angiotensin-converting enzyme 2 (ACE2) (A) and sNRP1 related proteins, sNRP1, VEGF, SEMA3A and ADAM17 (B) in T2D. No correlation of hyperglycemia with those proteins was found T2D subjects. Part A: Renin, open black circles; angiotensinogen (ANG), open blue squares; ACE2, open red diamonds. Part B: NRP1, open black circles; SEMA3A, open blue squares; ADAM9, open red diamonds; VEGF, open green triangles. [file Image_1.tif]

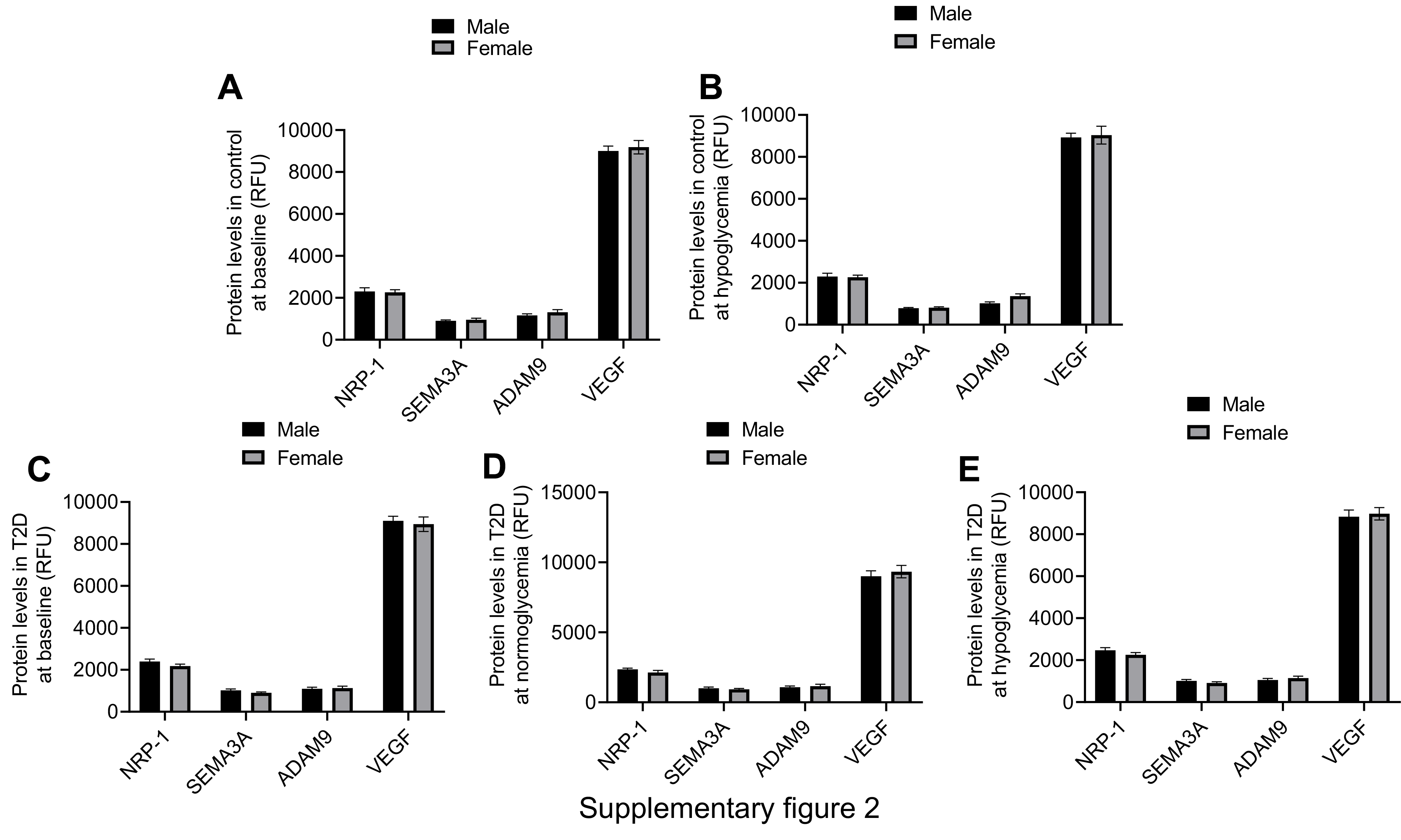

Supplement: Supplementary Figure 2 — Gender stratification of RAS proteins or sNRP1 related proteins at baseline or at hypoglycemia. No significant differences between male and female in the levels of sNRP1, VEGF, SEMA3A or ADAM17 at baseline and hypoglycemia in control (A, B); and at baseline, normoglycemia and hypoglycemia in T2D (C–E). [file Image_2.tif]
